# Annotation of Potential Vaccine Targets and Design of a Multi-Epitope Subunit Vaccine against *Yersinia pestis* through Reverse Vaccinology and Validation through an Agent-Based Modeling Approach

**DOI:** 10.3390/vaccines9111327

**Published:** 2021-11-15

**Authors:** Azaz Ul Haq, Abbas Khan, Jafar Khan, Shamaila Irum, Yasir Waheed, Sajjad Ahmad, N. Nizam-Uddin, Aqel Albutti, Nasib Zaman, Zahid Hussain, Syed Shujait Ali, Muhammad Waseem, Fariha Kanwal, Dong-Qing Wei, Qian Wang

**Affiliations:** 1 Center for Biotechnology and Microbiology, Kanju Campus, University of Swat, Swat 19200, Pakistan; azazulhaq612@gmail.com (A.U.H.); jafar@uswat.edu.pk (J.K.); nasibzaman@uswat.edu.pk (N.Z.); zahid@uswat.edu.pk (Z.H.); shujaitswati@uswat.edu.pk (S.S.A.); 2 Department of Bioinformatics and Biological Statistics, School of Life Sciences and Biotechnology, Shanghai Jiao Tong University, Shanghai 200240, China; abbaskhan@sjtu.edu.cn; 3 Department of Zoology, University of Gujrat, Punjab 50700, Pakistan; shamaila.irum@uog.edu.pk; 4 Multidisciplinary Department, Foundation University Medical College, Foundation University Islamabad, Islamabad 44000, Pakistan; yasir.waheed@fui.edu.pk; 5 Department of Health and Biological Sciences, Abasyn University, Peshawar 25000, Pakistan; sahmad@bs.qau.edu.pk; 6 Biomedical Engineering Department, HITEC University, Taxila 47080, Pakistan; nizam.uddin@hitecuni.edu.pk; 7 Department of Medical Biotechnology, College of Applied Medical Sciences, Qassim University, Buraydah 51452, Saudi Arabia; as.albutti@qu.edu.sa; 8 Faculty of Rehabilitation and Allied Health Science, Riphah International University, Islamabad 46000, Pakistan; m.waseem@riphah.edu.pk; 9 Med-X Research Institute, School of Biomedical Engineering, Shanghai Jiaotong University, Shanghai 200240, China; farihakaanwal@gmail.com; 10 Peng Cheng Laboratory, Vanke Cloud City Phase I Building 8, Xili Street, Nashan District, Shenzhen 518055, China; 11 State Key Laboratory of Microbial Metabolism, Shanghai-Islamabad-Belgrade Joint Innovation Center on Antibacterial Resistances, Joint Laboratory of International Cooperation in Metabolic and Developmental Sciences, Ministry of Education and School of Life Sciences and Biotechnology, Shanghai Jiao Tong University, Shanghai 200030, China; 12 Department of Medicine, Nanjing Medical University, No. 140, Hanzhong Road, Nanjing 210029, China

**Keywords:** *Yersinia pestis*, vaccine targets, epitope mining, in silico cloning, immune simulation

## Abstract

*Yersinia pestis* is responsible for plague and major pandemics in Asia and Europe. This bacterium has shown resistance to an array of drugs commonly used for the treatment of plague. Therefore, effective therapeutics measurements, such as designing a vaccine that can effectively and safely prevent *Y. pestis* infection, are of high interest. To fast-track vaccine development against *Yersinia pestis*, herein, proteome-wide vaccine target annotation was performed, and structural vaccinology-assisted epitopes were predicted. Among the total 3909 proteins, only 5 (rstB, YPO2385, hmuR, flaA1a, and psaB) were shortlisted as essential vaccine targets. These targets were then subjected to multi-epitope vaccine design using different linkers. EAAK, AAY, and GPGPG as linkers were used to link CTL, HTL, and B-cell epitopes, and an adjuvant (beta defensin) was also added at the N-terminal of the MEVC. Physiochemical characterization, such as determination of the instability index, theoretical pI, half-life, aliphatic index, stability profiling, antigenicity, allergenicity, and hydropathy of the ensemble, showed that the vaccine is highly stable, antigenic, and non-allergenic and produces multiple interactions with immune receptors upon docking. In addition, molecular dynamics simulation confirmed the stable binding and good dynamic properties of the vaccine–TLR complex. Furthermore, in silico and immune simulation of the developed MEVC for *Y. pestis* showed that the vaccine triggered strong immune response after several doses at different intervals. Neutralization of the antigen was observed at the third day of injection. Conclusively, the vaccine designed here for *Y. pestis* produces an immune response; however, further immunological testing is needed to unveil its real efficacy.

## 1. Introduction

*Yersinia pestis* is an etiologic agent of plague, which is infamous for three major pandemics killing millions of people [1]. Of these, the major devastating pandemic occurred in Europe, affecting a significant portion of the population [2]. The pathogen also has excellent potential for use in bioterrorism [3]. *Y. pestis* is a Gram-negative bacterium that transfers from fleas to humans and rodents [1]. Experimental studies performed on mice showed that after incubation of *Y. pestis* in mice, high growth of the pathogen was evident, leading to death of the mice in a few days [4]. It was noticed that *Y. pestis* caused inflammation and affected the function of the immune system [5]. This pathogenicity is the outcome of contributions from lipopolysaccharides (LPSs) that hold vital importance in tackling the host immune system [6]. The pathogen has two different plasmids that encode a number of virulent proteins. One is the pPCP1 plasmid, which encodes an activator of virulence essential plasminogen (Pla). The second pMT1 plasmid codes for F1 capsular protein and murine toxin Ymt, which are significant in plague infection transmission. Ymt encodes murine toxin and is lethal but required for its survival in fleas [6]. Furthermore, *Y. pestis* has machinery that inhibits MAPK pathway activation and the release of the proinflammatory cytokine TNF-α, according to recent findings. The same study also found that two *Y. pestis* strains, the fully virulent Kimberley53 and the pgm attenuated EV76 strain, are less capable of inducing macrophage apoptosis, impairing NF-B activation, and activating caspase pathways than the virulent *Y. enterocolitica* O:8 strain under varied infection circumstances. These limitations correlate with inefficient translocation of YopJ by *Y. pestis*, which leads to slow accumulation of the effector in the target cells. In addition, they also found that *Y. enterocolitica*-derived YopP can provide effective apoptotic potential to *Y. pestis*, and they suggested that this can be attributed to the fact that *Y. enterocolitica* O:8 YopP is better adapted to translocation by the *Yersinia* TTSS than YopJ [7].

*Y. pestis* interacts with Toll-like receptor (TLR) immune cells. TLRs are receptors present on human innate immune cells. TLRs can detect a large number of pathogenic molecules called PAMPs (pathogen-associated molecular patterns) on the bacterial cell wall components, LPS (lipopolysaccharide), and nucleic acid [8]. TLRs activate the hosts’ initial line of protection against the pathogen. Besides LcrV, *Y. pestis* has LPS as a major component in its outer membrane. This works as a ligand for Toll-like receptor 4 (TLR-4) and plays a basic role in virulence; this is why we used TLR-4 for our study [9].

In traditional vaccinology, microbes are cultured, followed by pathogenic antigen identification, characterization, isolation, inactivation, and reinjection into the host to provoke immune responses [10]. These traditional methods are expensive and time-consuming, and also not appropriate for pathogens like *Y. pestis.* Advancements in system biology, DNA sequencing, genomics, and proteomics have facilitated a better understanding of organism pathogenicity, contributing multifold to vaccine design. Such techniques have good accuracy and cost-effectiveness and have been successfully applied to many pathogens in the recent past with promising results [11,12,13,14,15,16,17,18]. Several types of vaccines based on the strategy of epitope predictions have been developed against human pathogens. This includes the development of a potential epitope (AMA-1) based vaccine candidate against *Plasmodium vivax* [19]. Recently, in 2019, a computationally predicted multi-epitope candidate vaccine was evaluated against *Acinetobacter baumannii* with potent IgG antibody-specific immune response in mice [20]. Similarly, the utility of computationally predicted B-cell epitopes was also confirmed in diagnostics against *Trypsonoma vivax* [21]. For instance, such approaches have been deployed against several human pathogenic viruses, including Ebola virus [22], Marburg virus [23], Crimean–Congo hemorrhagic fever virus [24], and Mokola Rabies virus [25]. Moreover, *Ziwei* et al. predicted T-cell and B-cell epitopes against SARS-CoV-2 and tested them experimentally, which resulted in a highly immunogenic response in experimental mice [26]. This reflects the potential implications of reverse-vaccinology-based approaches in vaccine design against human pathogenic viruses.

Keeping in view the importance of subtractive proteomics in vaccine target mining and reverse-vaccinology-based vaccine design, in the present study, we also aimed to design a multi-epitope subunit vaccine for *Y. pestis* by screening the whole proteome. Using subtractive proteomics followed by a reverse vaccinology pipeline, potential virulent and highly antigenic proteins from the *Y. pestis* proteome to design B-cell, HTL, and T-cell epitopes were identified. These epitopes were used in the design of a vaccine construct, which was then modeled and docked with TLR-4 immune receptor and subjected to biophysical analysis. Overall, the results presented in this study are interesting and may be useful to accelerating the development of an effective vaccine to combat this deadly pathogen.

## 2. Materials and Methods

### 2.1. Retrieval of Proteomes

The study was initiated by retrieving *Yersinia pestis’* proteome from UniProt, available under accession number UP000000815 (Consortium, 2014) [27].

### 2.2. Removal of Homologous Proteins

*Yersinia pestis’* proteins that are homologous to the human proteome in structure and function generate autoimmunity and cross-reactions; thus, they are not desired in vaccine development. Therefore, all the proteins were used in NCBI’s BLASTp against the human reference genome by setting the E-value threshold to 0.000001 [28].

### 2.3. Removal of Paralogous Proteins

The non-homologous proteins were then used in redundancy checks to discard duplicated sequences in the proteome. For this, a CD-HIT server was used, keeping the cut-off value at 0.8 (80%). The output file containing only non-redundant proteins was analyzed further in the downward analysis [29].

### 2.4. Non-Essential Protein Removal

Essential proteins are vital for the survival of an organism, and their disruption leads to cell death. The Database of Essential Genes (DEG) contains all essential genes of viruses, bacteria, and fungi [30]. Therefore, the non-redundant set of proteins was checked via BLASTp against the DEG, and only proteins with hits (E-value 0.000001) were considered for further analysis. Proteins of small size (<100 amino acids) were manually removed from the essential proteome as they do not harbor potential epitopes.

### 2.5. Subcellular Localization of Proteins

Protein subcellular localization was performed to explore the locations of the essential proteins, as surface proteins of bacterial cells are exposed and easily recognized by the host immune system. We used the CELLO online server, keeping the class bacteria and the type Gram-negative [31].

### 2.6. Prioritizing Potential Vaccine Candidates

We prioritized the cell membrane, peripheral membrane, extracellular, and outer membrane proteins for subunit-based vaccine design. On the contrary, cytoplasmic proteins contain hydrophobic pockets for binding to small drug molecules; thus, we removed the cytoplasmic proteins from the vaccine design perspective [17,18,32].

### 2.7. Collection of Virulent Proteins

Virulent proteins have pathogenic characteristics like adherence to the host cells, invasion, toxin action, and actin-based motility, and they are involved actively in secretion systems [33]. The Virulence Factor Database (VFD) was used to predict the virulent propensity of the proteins filtered in the previous step using PSI/PHI BLAST, and those with >30% identity and >100% bit score were allowed to enter the next phase [34].

### 2.8. Antigenicity of the Proteins

Antigenicity is the capacity of proteins (epitopes) to bind with immune cells and generate productive adaptive immune responses [35]. The capacity of a foreign particle (antigen) to attach to or interact with the products of the final cell-mediated response, such as B-cell or T-cell receptors, is referred to as “antigenicity”. Antigenicity is the term used to define whether and how well the substance attaches to immune cells, beginning the immune response process. Antigenicity may also be described as the ability of an antigen or a hapten to bind to a B-cell’s or T-cell’s receptors. To trigger an immune response, an antigen binds to the receptor of an immune cell, such as a B-cell or T-cell. The surface of every antigen or hapten has a unique biological key called an epitope. It is this epitope that binds with a corresponding receptor or antibody. When T-cell receptors interact with a major histocompatibility complex (MHC) molecule, they identify linear amino acid sequences inside a protein antigen, commonly known as epitopes. Hence, it is important to estimate the antigenic potential of each protein and their potential epitopes. The antigenicity of protein was checked via the VaxiJen server at 0.4 threshold [36].

### 2.9. Prioritization of Proteins

Five proteins (rstB, YPO2385, hmuR, flaA1, and psaB) were prioritized. These proteins have a length of more than 100 amino acids, are virulent and antigenic in nature, and have a molecular weight of more than 100 kbs.

### 2.10. Epitope Prediction

The five selected proteins, rstB, YPO2385, hmuR, flaA1, and psaB, were used for the prediction of epitopes. Cytotoxic T lymphocyte (CTL) and helper T lymphocyte (HTL) epitopes were predicted by using two different servers. For CTL epitope prediction, the NetCTL 1.2 server was used, and HTL epitopes were predicted by the IEDB MHC II server [37]. Epitopes from each protein were selected on the basis of the highest combined score among selected epitopes. The E-value was set to 0.75. Seven alleles were selected as an HLA reference set. Epitopes of 15mer in size were selected, three from each protein. The AlgPred server determined the allergenicity of each HTL epitope.

### 2.11. Vaccine Construct Design

A final vaccine construct was designed by linking the epitopes at a ratio of 2:3 CTL and HTL, comprising 8 CTL and 12 HTL. Overlapping HTL epitopes were not used in the construct design. Adjuvant was joined to the CTL epitope peptide at the N-terminal using the EAAAK linker, whereas CTL epitopes were fused by the AAY linker. The last CTL epitope was joined to HTL by a GPGPG linker.

Ratio: 2:3

2 × 4 = 8 CTL epitopes

3 × 4 = 12 HTL epitopes

### 2.12. Allergenicity Prediction

The vaccine construct was further scanned for allergenic sequences by analyzing it in the AlgPred 2.0 server (https://webs.iiitd.edu.in/raghava/algpred2/, accessed on 30 December 2020) [38]. The AlgPred server uses different algorithms such as SVM + MAST + ARPs BLAST + IgE epitope to properly evaluate allergenic sequences with up to 85% accuracy.

### 2.13. Antigenicity Evaluation

The antigenic nature of the vaccine is very important and is required to boost the immune system. The antigenicity of the vaccine construct was evaluated by using a freely accessible server, VaxiJen v 2.0. The accuracy of this server varies from 70% to 80%.

### 2.14. Evaluation of Physiochemical Parameters

The final vaccine construct was analyzed further in the ProtParam server to estimate its instability index, theoretical pI, half-life, aliphatic index, stability profiling, and hydropathy; the Grand Average and other properties were calculated [39].

### 2.15. Homology Modeling of the Vaccine Construct

The three-dimensional (3D) model of the constructed vaccine was modeled using the Robetta (http://robetta.edu/StructurePrediction/predict/ (accessed on 05 January 2021)) webserver. Robetta is good at predicting query protein 3D structures without close similarity (<30%) of the input sequence [40]. A total of five models were generated, and the one with the best prediction score was chosen. The vaccine construct secondary structure was predicted by the PSIPRED 3.0 server [41].

### 2.16. Tertiary Structure Refinement

The tertiary structure of the vaccine was refined using GalaxyRefine to improve its local and global structural quality [42].

### 2.17. Structure Validation

The 3D structure of the vaccine construct was then validated by using three freely available online servers: RAMPAGE (http://mordred.bioc.cam.ac.uk//~rapper/rampage.php, accessed on 05 January 2021) [43], ProSA-web, accessed on https://prosa./services.came.sbg.ac.at/prosa.php, accessed on 05 January 2021) [44], and ERRAT (http://servicesn.mbi.ucla.edu//ERRAT/, accessed on 05 January 2021) Lengths and Angles [45].

### 2.18. B-Cell Epitope Prediction

B-cell epitopes trigger humoral immunity to produce antibodies specific to the antigen, and memory cells are significant in memorizing the pathogen and responding on re-encounter [46]. Linear and conformational B-cell epitopes were mapped for the subunit vaccine by using the B-cell epitope prediction server of BCPREDS [47]. Conformational B-cell epitopes were predicted by IEDB MHC Ellipro [48].

### 2.19. Molecular Docking of the Vaccine Constructs and TLR-4

Molecular docking was used to predict the binding conformation and interactions of the vaccine construct with the TLR-4 immune receptor. Docking was performed using a blind docking approach through the HAWKDOCK server [49]. The server functions by using a geometry-based molecular docking algorithm that gives the best docked intermolecular conformation [49].

### 2.20. Molecular Dynamics Simulation

A molecular dynamics simulation of the vaccine–TLR complex was performed to check the stability of the docked complex. The AMBER20 simulation package was used [50]. Two steps of energy minimization followed by heating, equilibration, and production were performed. A total 100 ns simulation was performed. Default parameters were used as given in the previous study [11,13,51]. The *RMSD* and *RMSF* were calculated to check the binding stability and flexibility.

(1) $$\mathrm{RMSD}=\sqrt{\frac{\sum_{i=0}^{N}{[m}_{i}{*(X}_{i}*\;Y_{i}{)}^{2}]}{M}}$$

Here, *N* is the number of atoms, *m_i_* is the mass of atom *i*, x*_i_* is the coordinate vector for target atom *i*, *Y_i_* is the coordinate vector for reference atom *i*, and *M* is the total mass. If the *RMSD* is not mass-weighted, all *m_i_* = 1 and *M* = *N*.

The *RMSF* is a measure of the deviation between the position of particle *i* and some reference position:

$$RMSF_{i}=\left|\begin{array}{c} 1\\ T \end{array}\overset{T}{\underset{t_{j}=1}{\sum }}{\left|r_{i}\left(t_{j}\right)-r_{i}{}^{ref}\right|}^{2}\right|1/2$$

where *T* is the time over which one wants to average and *ri^ref^* is the reference position of particle *i*. This reference position will be the time-averaged position of the same particle *i*.

### 2.21. Codon Optimization and Cloning of the Vaccine Construct

The codon usage of the vaccine construct was adjusted according to the *Escherichia coli* system to obtain better expression of the vaccine sequence. For this, Jcat software was used first to generate a reverse transcript of the input vaccine sequence, with calculation of the GC content. An appropriate CAI value (needs to be 0.9+) and GC content (45–70%) are indications of a well-optimized sequence [52]. To carry the vaccine sequence into the expression system, suitable restriction enzyme sites were created to ease the insertion of the vaccine into the vector. SnapGene software was used for cloning purposes [53].

### 2.22. Immune Simulation

In order to understand the dynamics of the human immune system in response to foreign particles, a server that uses agent-based modeling, C-ImmSim, was used to predict the relationships between the human immune system and the foreign particle [54]. The production of cytokines and other substances like interferon and antibodies was estimated by applying the PSSM method. Moreover, the response for T helper cell 1 and T helper cell 2 (Th1 and Th2) was also predicted with the server’s default parameter measure of diversity or Simps Index [55]. The immune simulation uses the following mathematical model to estimate the interaction of the vaccine and therapeutics.

$$\frac{dT}{dt}=rT-kTE$$

$$\frac{dE}{dt}=f\left(T\right)+g\left(E\right)-dE$$

The above linear mathematical model was used to model the immune simulation of the designed vaccine [56].

## 3. Results

### 3.1. Retrieval of Proteomes

**Figure 1:**
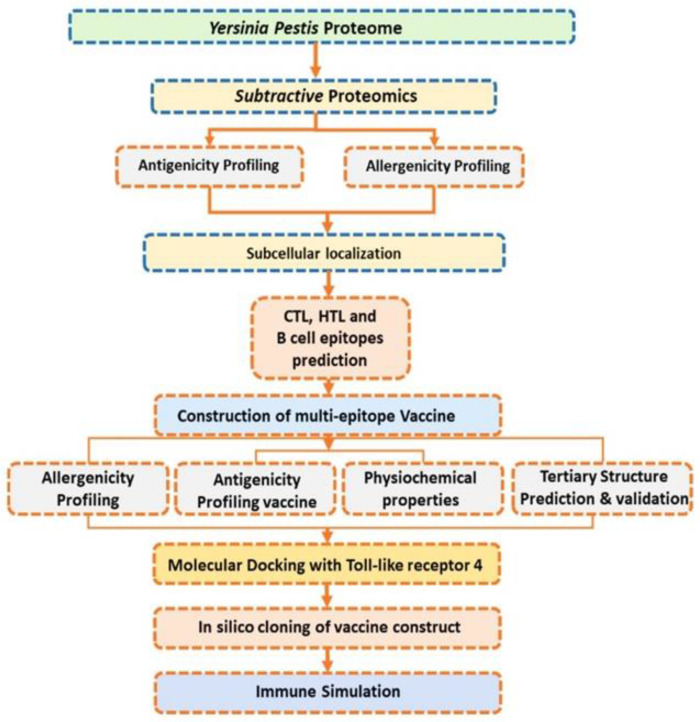
Workflow of the complete methodology, shown in eight steps. First, the selected proteins’ antigenicity was checked. Secondly, virulence proteins were screened, giving five in number. The third step was B- and T-cell epitope prediction; the fourth step was the construction of the vaccine; the fifth step was determining the different physicochemical parameters of the vaccine construct, the sixth step was molecular docking; the seventh step was in silico cloning; and the eighth and last step was an immune simulation.

The pathogen proteome comprises a total of 3909 proteins. These proteins were subsequently subjected to the designed vaccine framework to prioritize potential vaccine targets and design a vaccine ensemble. The whole methodological flow is shown in Figure 1.

### 3.2. Removal of Homologous Proteins

*Y. pestis* proteins that were homologous to the human proteome were discarded from the complete proteome; thus, 1021 proteins were filtered as non-homologous. This set of proteins was considered for additional analysis as they are not able to elicit any non-specific responses.

### 3.3. Removal of Paralogous Proteins

In total, 704 proteins were found to be non-redundant and were therefore allowed into further steps of the vaccine design framework. These proteins are singly represented in the proteome and are critical to the pathogen’s core functional pathways.

### 3.4. Removal of Non-Essential Proteins

The essential proteins play a central role in pathogen survival and are good candidates for vaccine design. A total of 368 essential proteins were found after excluding non-essential and small-sized proteins.

### 3.5. Subcellular Localization

Subcellular localization analysis revealed 73 periplasmic, 46 outer membrane, 152 inner membrane, and 11 extracellular proteins. Such proteins come in close contact with the host during interactions and contain antigenic determinants in vaccine design.

### 3.6. Collection of Virulent Proteins

**Figure 2:**
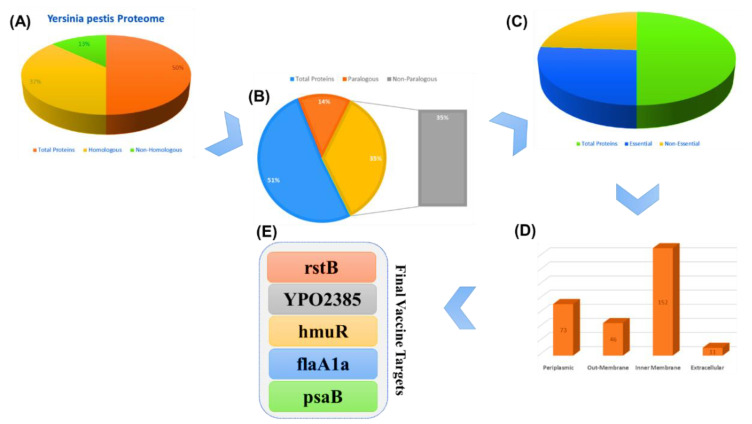
The steps of the subtractive proteomics, and the results obtained from each step. (**A**) represent the whole proteome of Y. pestis with the total number of proteins, homologous and non-homologous. (**B**) shows the whole proteome of Y. pestis with the total number of proteins, paralogous and non- paralogous. (**C**) whole proteome of Y. pestis with the total number of proteins, essential and non-essential. (**E**) final vaccine targets while (**D**) represent the cellular location of different proteins.

Virulent proteins stimulate infectious pathways of the host, allowing an efficient response from the immune system to the antigenic region of the proteins. The five selected proteins’ (rstB, YPO2385, hmuR, flaA1a, and psaB) amino acid sequences were retrieved from the gene bank by using their gene ids (1175141, 1175217, 1173129, 1173578, and 1174146) for the multi-epitope subunit vaccine design (Figure 2).

### 3.7. Antigenicity and Physiochemical Properties of the Selected Vaccine Targets

Antigenic proteins that have the capacity to bind with the immune cells’ receptors efficiently were predicted from among the virulent proteins. Among the total, only five highly antigenic proteins were selected, shown in Table 1. The physiochemical properties of the five selected vaccine targets (rstB, YPO2385, hmuR, flaA1a, and psaB) were then determined. These physiochemical properties, such as virulence, antigenicity, mass, and total number of amino acids, are given in Table 1.

### 3.8. CTL and HTL Epitope Prediction

Epitope prediction is an essential step in vaccine design as the immune system recognizes and binds to epitopes to activate specific immunity. In total, 72 CTL epitopes were predicted for the five selected proteins; these can be split into 14 epitopes for sensor kinase protein, 10 epitopes for putative exported protein, 26 epitopes for hemin receptor, 9 epitopes for flagellin, and 13 epitopes for chaperone protein PsaB. Only 8 CTL epitopes were selected because of their high MHC binding score and non-allergenic nature. The selected set of epitopes comprises two epitopes from sensor kinase protein, one from putative exported protein, two from hemin receptor, one from flagellin, and two from chaperone protein PsaB, as tabulated in Table 2.

A total of 12 HTL epitopes were selected, giving a 2:3 ratio; three of the HTL epitopes were selected from sensor kinase protein, two from putative exported protein, three from hemin receptor, and two each from flagellin and chaperone protein PsaB, as shown in Table 3.

### 3.9. B-Cell Epitope Prediction

B-cells, upon maturation, produce antibodies and are essential for long-lasting immunity against a particular antigen. In total, 16 linear B-cell epitopes with score >0.6 were selected. Likewise, the ElliPro suite for the prediction of conformational B-cell epitopes with a score of 0.7 was used. B-cell epitopes were predicted from the selected five proteins. A total of 17 epitopes were predicted from Chaperone protein, out of which 4 epitopes were chosen on the basis of a score above 0.9; a total of 24 epitopes were predicted from Histidine kinase, out of which 2 epitopes were selected. Twenty-eight epitopes were predicted from the Hemin receptor, out of which two were selected with a high score. From 24 epitopes predicted from Flagellin protein, 4 epitopes were chosen, and of 21 epitopes predicted from Putative exported protein, 5 epitopes were selected on the basis of a score above 0.9. The predicted linear B epitopes are given in Table 4.

### 3.10. Vaccine Construct Design

**Figure 3:**
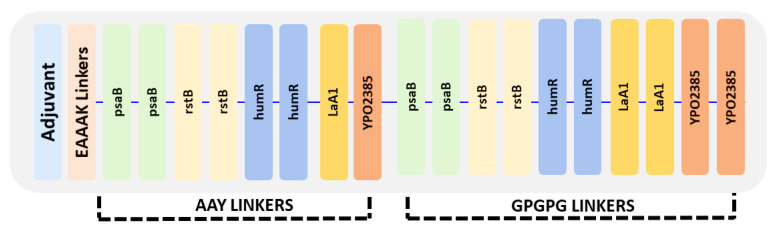
Arrangement of the final vaccine construct design comprising CTL and HTL epitopes using three different linkers: AAY, GPGPG, and EAAAK.

The vaccine ensemble was generated by joining epitopes at a ratio of 2:3; in total, 8 CTL and 12 HTL epitopes were added using linkers. The overlapping HTL epitopes were excluded during vaccine design. The adjuvant was joined to CTL epitopes by using EAAAK linkers at the N terminal, whereas CTL epitopes were joined via AAY linker. The last CTL epitope was joined with HTL by GPGPG linkers, and GPGPG linkers were also used among HTL epitopes, as given in Figure 3.

### 3.11. D Structure Prediction Using Robetta

**Figure 4:**
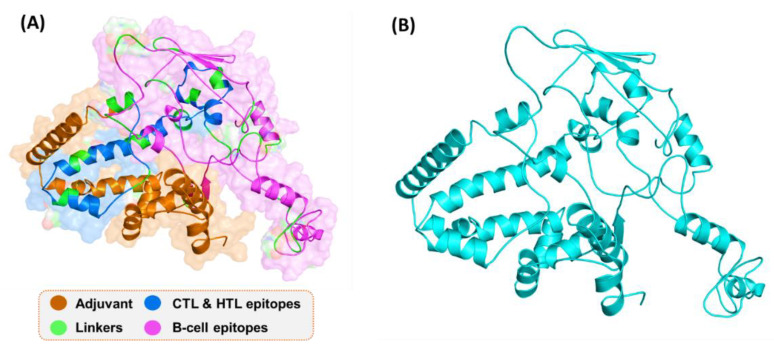
3D structure of the final vaccine construct. (**A**) The distribution of different peptides and linkers on the protein surface; (**B**) The 3D structure of the final vaccine construct.

By using the Robetta server, 3D structures of the vaccine were constructed. The modulated structure is shown in Figure 4. A comparative modeling approach was used, and multiple templates were selected for the 3D structure model design. The confidence levels of the templates for 3D structure design were all above 97.9%. The 3D structure predicted by Robetta, as shown, possesses alpha-helices, beta-sheets, and loops. The visual structure analysis shows that it has proper folding.

### 3.12. Refinement of the Vaccine Tertiary Structure

The results of refinement analysis of the vaccine are tabulated in Table 5. It was concluded that Model 2 was the best model for docking. Model 2 (Figure 4) had a high GDT-HA score of 0.9583, and its root mean square deviation value was 0.402. The MolProbity score was 1.400, the clash score was improved, poor rotamers were 0.6, and the Rama favored level was calculated to be 98.5%.

### 3.13. Vaccine 3D Structure Validation

**Figure 5:**
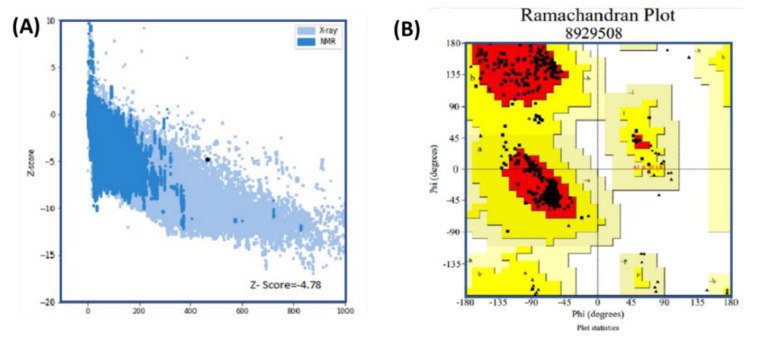
Results of analysis using the Ramachandran and PROSA-web servers. Panel (**A**) shows the Z-score (4.78) predicted by PROSA-web. In panel (**B**), the Ramachandran plot shows the amino acid distribution in the favored, allowed, and disallowed regions. Favored regions are reported with 91.8% residence, with 7.9% allowed and 0.3% reported as being disallowed.

The 3D structure was validated using three available online servers. The results of analysis using the Ramachandran and PROSA-web servers are given in Figure 5. Panel (A) shows the Z-score (−4.78) predicted by PROSA-web, while in panel (B), the Ramachandran plot shows the amino acid distribution in the favored region as 91.8%, with 7.9% allowed and 0.3% disallowed. These results suggest that our predicted structure has a properly folded topology and could be used for further processes.

### 3.14. Physiochemical Properties of the Final Vaccine

The vaccine construct is non-allergenic with a score of −1.52; isoelectric point of 9.34; molecular weight of 49.70 kDa; half-life of >30 h (mammalian reticulocytes, in vitro), >20 h (yeast, in vivo), or >10 h (*E. coli*, in vivo); instability index of 35.03; GRAVY score of −0.225; and aliphatic index of 73.14. All these parameters strongly support the construct as a good vaccine candidate. From an antigenicity point of view, the vaccine is a good antigen (score 0.7548). The vaccine molecule is also non-allergic (score 1.523).

### 3.15. Secondary Structure Prediction

**Figure 6:**
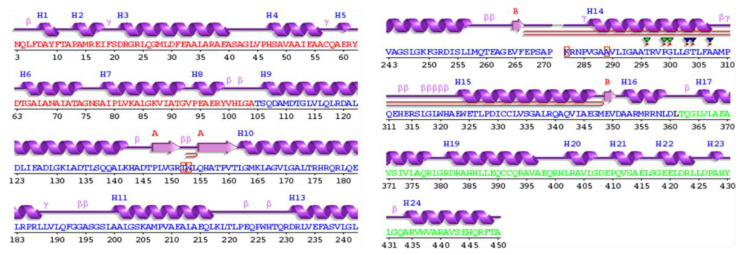
Secondary structure of the vaccine, which shows 35.7% helices, 14.3% B sheets, and 50% coils.

The PSIPRED webserver was used to predict the secondary structure of the vaccine construct shown in Figure 6. The secondary structure of the vaccine revealed 35.7% alpha helices (including 4% transmembrane helices), 14.3% beta sheets, and 50% coils.

### 3.16. Analysis of Vaccine Construct Interaction with TLRs

**Figure 7:**
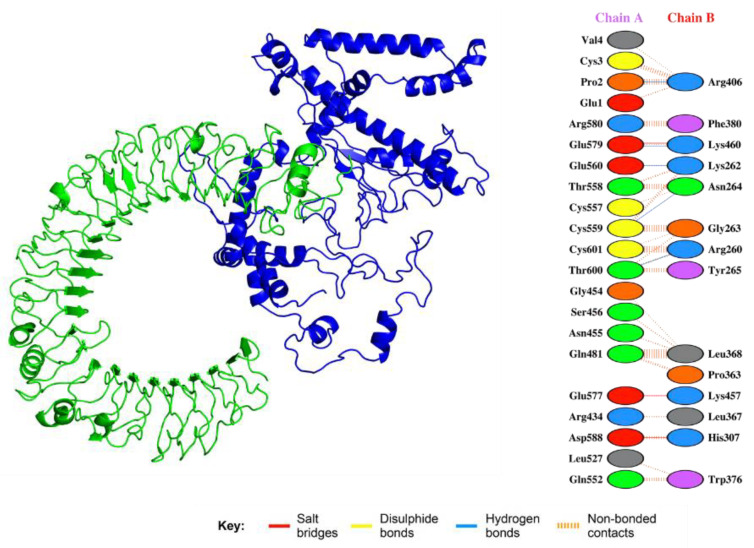
Interaction between the vaccine and TLR. The green represents the TLR, while the blue color shows the multi-epitope vaccine construct. Specific interactions are given in the right-hand panel. Salt bridges, disulphide bonds, and hydrogen bonds are shown in different colors.

Solution 2 of vaccine–TLR-4 was selected as the best-docked complex. The selection was based on FireDock results with global energy of −15.47, attractive van der Waals force of −11.27, repulsive van der Waals force of −4.39, and atomic contact energy of −3.98. The interactions given in Figure 7 show that the vaccine candidate is significantly involved in salt bridges, hydrogen bonding, and disulfide bridges.

### 3.17. Molecular Simulations of TLR–Vaccine Complexes

**Figure 8:**
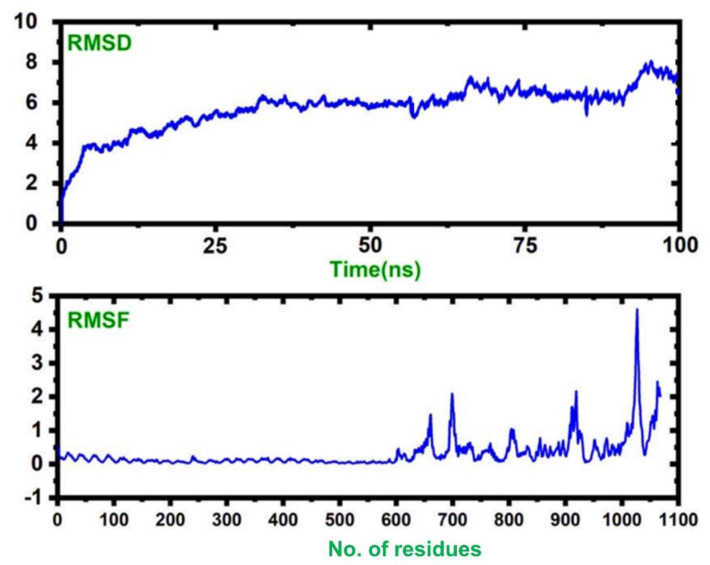
The dynamic stability, given as the RMSD, and the residual flexibility, given as the *RMSF*.

A molecular dynamics simulation of the vaccine–TLR complex revealed that the complex is stable with no significant convergence, while the *RMSF* results show that the vaccine structure possesses higher flexibility than the TLR. Our results suggest that our complex remained stable during the 100 ns simulation, and this signifies our vaccine’s efficiency. The *RMSD* and *RMSF* values of the complex are given in Figure 8.

### 3.18. Codon Optimization and Cloning of the Vaccine Construct

**Figure 9:**
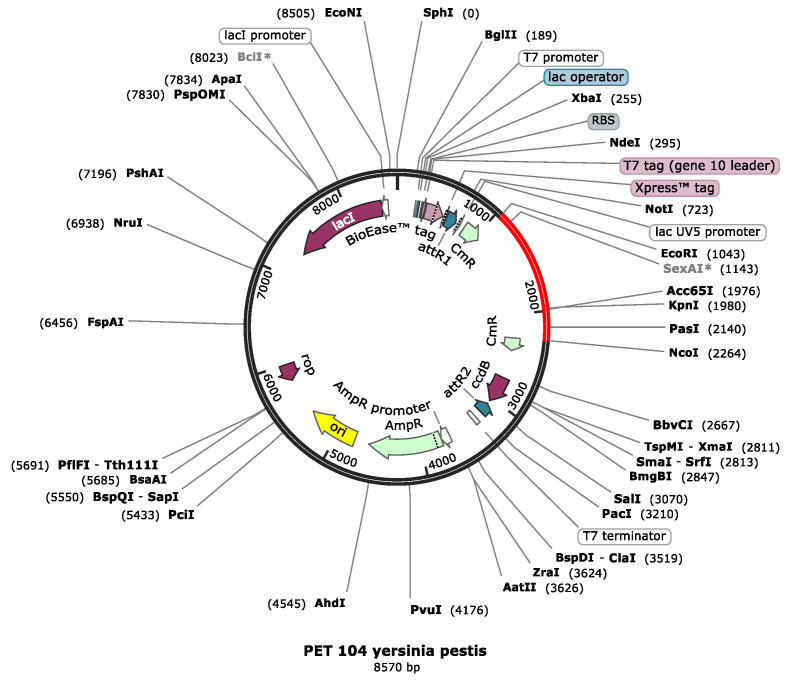
The pET-104 vector along with the vaccine construct inserted between the restriction sites with ECOR1 upstream and NCO1 downstream.

JCat software was utilized to quantify the expression of vaccine in *Escherichia coli* O6: k15:H31 (strain 536/UPEC). The vaccine is 1400 nucleotides long; its CAI is 1.0 with a GC content of 55.19%. This indicates a better expression level of the vaccine in *E. coli* sO6: k15:H31 (strain 536/UPEC). A GC content range of 35% to 70% is reported to be appropriate for better expression. Restriction sites like EcoR1 and Pas1 were added to the 5′ and 3′ ends of the optimized vaccine sequence and cloned in pET-53-Dast vector as given in Figure 9.

### 3.19. Immune Simulation

**Figure 10:**
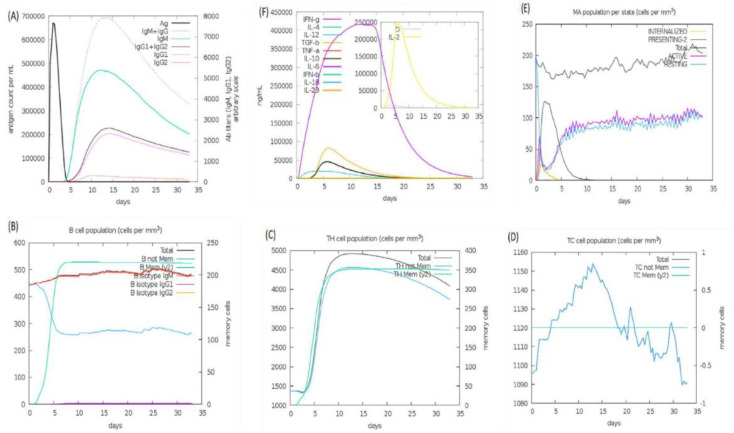
Immune simulations: panel (**A**) shows the number of immunoglobulin reactions per milliliter of antigen, panel (**B**) shows the count of B lymphocytes, panel (**C**) shows the count of CD4 T-cells, panel (**D**) shows the count of CD8 T-cells, panel (**E**) shows the macrophages and the amount of internalized pathogens, and panel (**F**) shows the secretion concentrations of cytokines and interleukins.

An immune simulation study was performed on web server *C-ImmSim* 1.0, which predicts possible dynamics of the host immune system in response to vaccine administration. The host immune system dynamics are summarized in Figure 10. Panel A shows the humoral immunity response to the antigen and reported immunoglobulin response: IgG, IgG1, IgG2, and IgM produced against the vaccine. According to the literature, most plague patients have been reported with igG1 and IgM. In parts B, C, and D, production of B- and T-cells in cells per cubic millimeter is shown to be very high in the presence of vaccine; the B-cell concentration is high and remains so because they produce antibiotics, staying active for a longer time. The graph for the T-cells shows a downward trend after a few days. Panel E shows macrophages, and panel F shows the interleukin and cytokine concentration, which was very high for the vaccine inside immune cells for the first few days.

## 4. Discussion

For infectious disease, antibiotic controls are traditionally considered; however, due to their decreasing effectiveness, the scientific community has begun the search for a new approach, including vaccines that could replace antibiotics. Vaccinations were initially used for infectious disease control and played a significant role in this, but recently, due to our greater dependency on them, scientists have sought more effective, cheap, and safe techniques for designing vaccines. Because traditional vaccine technology harbors the risk of virulence reoccurrence and is not very cost-effective, a new technology, called immune-informatics, has been developed, requiring knowledge of genomics and proteomics to bring new vaccines to the market. This uses only a particular part of the pathogen (epitope) which can only boost the immune system. Immune informatics technology uses computational software, online servers, and databases. It gives good-quality results and is low cost, with good safety and greater effectiveness. Several types of vaccines based on the strategy of epitope predictions have been developed against human pathogens. This includes the development of a potential epitope (AMA-1) based vaccine candidate against *Plasmodium vivax* [19]. Recently, in 2019, a computationally predicted multi-epitope candidate vaccine was evaluated against *Acinetobacter baumannii* with potent IgG antibody-specific immune response in mice [20]. Similarly, the utility of computationally predicted B-cell epitopes was also confirmed in diagnostics against *Trypsonoma vivax* [21]. For instance, such approaches have been deployed against several human pathogenic viruses, including Ebola virus [22], Marburg virus [23], Crimean–Congo hemorrhagic fever virus [24], and Mokola Rabies virus [25]. Moreover, *Ziwei* et al. predicted T-cell and B-cell epitopes against SARS-CoV-2 and tested them experimentally, which resulted in a highly immunogenic response in experimental mice [26]. This reflects the potential applications of in silico reverse-vaccinology-based approaches in vaccine design against human pathogenic viruses.

The proteome is the protein factory of an organism and contains the proteins of traits present in an organism. This is why we exploited the proteome of *Yersinia pestis* to design a vaccine. Highly antigenic and immunogenic proteins rstB, YPO2385, hmuR, flaA1, and psaB were selected using online servers, and the epitopes for adaptive immune cells, B lymphocytes, and T-cell lymphocytes were predicted. A foreign organism first deals with innate immune cells, so we added adjuvants that interact with the TLR of innate immune cells. Epitopes were predicted through online servers, which showed the binding potency of the epitopes with MHC-I and MHC-II of T-cells, and predicted B-cell linear epitopes that interacted with immune B-cells. The designed vaccine’s physiochemical properties were evaluated. Although the designed vaccine candidate exhibits non-allergenic features, the “cytokine storm” phenomenon cannot be avoided in infectious diseases, but this is rare. Based on recent developments in the field, new therapies will likely move away from targeting single soluble mediators and focus more generally on inflammatory cascades. However, what signaling cascades are targeted will depend both on the infection and on the targeted patient population. Current experimental anti-inflammatory strategies being tested in animal models include COX inhibitors, PAR2 agonists, sphingosine 1 phosphate modulators, modulation of production/signaling of oxidized phospholipids, Slit-Robo4 pathway modulators, and TLR-4 antagonists. These anti-inflammatory agents can be supplied as supplementary agents to overcome the problem of cytokine storm [57].

Furthermore, the 2D structure was designed using the PSI PRED server, and the 3D structure was predicted by the Robetta server and refined by the GALAXY refinement server. Further corrections were made using Chimera software. Then, more of the interaction with the TLR of innate immune cells was checked by docking analysis using PatchDock. Stability and fluctuations were achieved by MD simulation.

High levels of expression were needed. Thus, the Jcat server was used as it automatically performs reverse transcription, CAI calculations, and calculations of GC contents for codon optimization. E. coli was used as an expression vector. Vaccine stability is required for better expression in *E. coli*; therefore, a bisulfate bond was added with the vaccine construct.

Immuno-informatics approaches were used in the present study to give a stable, very effective, and highly expressible vaccine in the *E. coli* expression vector. Clinical trials are required to check its effectiveness in human beings. The designed MEVC construct can be further used in an experimental setup to evaluate the in vitro and in vivo potency, safety, and efficacy of the vaccine construct.

## 5. Conclusions

The plague is one of the deadliest diseases remaining in the present day, and the agent of this disease is *Yersinia pestis*. In the study presented herein, we used immune informatics techniques to design a vaccine from the epitopes of *Yersinia pestis* for better control. Such a vaccine is capable of providing immunity for both types of immune systems. Different techniques were used, such as molecular docking, simulation for thermal stability, and in silico cloning, to verify its thermal stability inside the human body, its better expression in vectors for mass production, and its immune response boost inability. The current study has the only limitation of lacking experimental testing, which can be performed by experimental scientists to confirm the validity of the designed candidate and its use for therapeutic purposes.

## Figures and Tables

**Table 1:** The genes, protein names, antigenicity scores, virulence scores, molecular masses, and amino acid lengths of the five shortlisted vaccine targets.

Gene	Protein	Virulence (Bit Score)	Virulence Sequence Identity	Antigenicity	Mass	Length
rstB	Sensor kinase protein	182 bits	33%	0.6925	48,250 Da	425
YPO2385	Putative exported protein	61.2 bits	34%	0.7901	32,315 Da	288
hmuR	Hemin receptor	886 bits	68%	0.6496	74,230 Da	676
flaA1	Flagellin	97.4 bits	40%	0.7208	42,701 Da	404
psaB	Chaperone protein PsaB	531 bits	100%	0.6251	30,648 Da	273

**Table 2:** The shortlisted CTL epitopes for each protein with the combined scores predicted by NetCTL server.

Protein Name	Epitopes	Sequence	Combined Score
Sensor kinase protein	CTL	RTPLVRLRY	3.1800
	CTL	MAMLVGLVY	3.1360
Putative exported protein	CTL	CSGLIYYAY	2.6220
Hemin receptor	CTL	QSLSANLRY	3.0050
	CTL	SSSTPQAGY	3.0610
Flagellin	CTL	ETPKAVNEY	2.6930
Chaperone protein PsaB	CTL	SADSLTWRY	2.7150
	CTL	PTPFYMNFY	2.5490

**Table 3:** Helper T lymphocyte (HTL) epitopes predicted by the IEDB MHC 2 server, along with their percentile ranks, alleles, peptide sequences, and antigenicity scores predicted by the AlgPred server.

Protein	Allele	Start–End	Peptide Sequence	Method	Percentile Rank	Antigenicity Score
Sensor kinase protein	HLA-DRB5 * 01:01	142–156	LPVFLWMRPHWKDLL	Consensus (smm/nn/sturniolo)	0.6	0.432
	HLA-DRB3 * 02:02	404–418	GGASFRFSWPIKTHL	NetMHCIIpan	1.4	1.043
	HLA-DRB1 * 03:01	362–376	EPFVRLDPSRDRATG	Consensus (smm/nn/sturniolo)	1.6	0.564
Putative exported protein	HLA-DRB3 * 02:02	207–221	NEMYHLRDAAPVKRT	NetMHCIIpan	5.1	1.042
	HLA-DRB5 * 01:01	161–175	AMSKLMKQVGKPYRW	Consensus (smm/nn/sturniolo)	9.7	0.613
Hemin receptor	HLA-DRB1 * 03:01	283–297	RSTIQRDAQLRYNIK	Consensus (smm/nn/sturniolo)	0.14	0.443
	HLA-DRB1 * 07:01	340–354	NRTRLFIESPASHLL	Consensus (comb.lib./smm/nn)	0.54	0.480
	HLA-DRB1 * 15:01	434–448	TDWLMLFGSYAQAFR	Consensus (smm/nn/sturniolo)	0.86	0.679
Flagellin	HLA-DRB1 * 07:01	25–39	NAKSSQRLSTGFRIN	Consensus (comb.lib./smm/nn)	3.1	0.461
	HLA-DRB1 * 15:01	382–392	QSSVMMLKKANAATQ	Consensus (smm/nn/sturniolo)	7.9	−0.444
Chaperone protein PsaB	HLA-DRB5 * 01:01	89–103	APFIVTPPLFRLDAG	Consensus (smm/nn/sturniolo)	0.77	0.722
	HLA-DRB1 * 15:01	181–195	ADSLTWRYKGNYLEV	Consensus (smm/nn/sturniolo)	4.2	0.504

**Table 4:** Predicted B-cell epitopes, along with their scores and sequence information.

Protein	Sequence	Starting Position	Score
Chaperone protein PsaB	APFIVTPPLFRLDAGL	89	0.95
	CLTGIPPKNGDAWGNT	128	0.94
	YPSSSTKGVSVSVANP	54	0.91
	SLTWRYKGNYLEVNNP	183	0.90
Histidine kinase	SWPIKTHLPLSADQNV	411	0.94
	SGHLDERTHFDPTSSL	167	0.91
YERPE Hemin receptor	PVSILAGTRYDNYSGS	396	0.94
	YETVDAADMLQPGQNS	164	0.93
	KDYISTRVDMQAMTTT	520	0.92
	SRVSSSTPQAGYGVND	612	0.91
	NWDLAYNRTRGKNQNT	559	0.91
	TRDIGNIRQSNGFNAP	215	0.91
	GLTLTNYWVPNPNLKP	469	0.90
	PTMGEMYNDSKHFAIP	450	0.90
	SGSSDGYADVDADKWS	409	0.90
	GWLQDEITLRDLPVSI	384	0.90
	ARPQGSAEEGREQTTE	319	0.90
YERPE Flagellin	SDVIDAYGAFRATLGA	321	0.95
	GFRINSPADNAAGLQI	35	0.93
	LGSIKDTDFADEMKNH	359	0.92
	KQEIETPKAVNEYVVK	280	0.92
	AESVKTLNAMKKLATQ	359	0.91
Putative exported protein	HVSQASPDDRKKRKAD	41	0.96
	GPVSKKTTEPRKTGNN	69	0.93
	SGLIYYAYKDVVKIKM	187	0.92
	GKFIQSPRTGEEIRIS	249	0.91
	TSSIRTAKTPYGRQRN	113	0.9

**Table 5:** The tertiary structure of the vaccine was refined using the GalaxyRefine server, and Model 2 was selected due to its good Rama favored score of 98.54 and low mol probity of 1.40.

Model	GDT-HA	RMSD	MolProbity	Clash Score	Poor Rotamers	Rama Favored
**MODEL 1**	0.949	0.42	1.42	7.81	0.62	98.31
**MODEL 2**	0.958	0.40	1.40	7.31	0.61	98.54
**MODEL 3**	0.948	0.42	1.46	8.72	0.92	98.32
**MODEL 4**	0.961	0.39	1.46	8.63	0.64	98.52
**MODEL 5**	0.943	0.42	1.46	8.61	0.66	98.33

## Data Availability

All the data will be provided on reasonable request.
